# 

**Published:** 1825-10-01

**Authors:** 


### Contents

OF

No. 6. Vol. III. (NEW SERIES) for OCTOBER, 1825.

[*No.* 22, *ANALYTICAL SERIES.*]

Art. I.

Histoire des Marais, et des Maladies causées par les Emanations des Eaux Stagnantes. Par J. B. Monfalcon, M.D. &c. .. 317

II.

Observations on Extraction of Diseased Ovaria; illustrated by Plates. By John Lizars, Surgeon, &c. &c. .. .. .. .. 335

III.

Dr. Ratier on the Hospital Practice of Paris. (Concluded from page 111.) .. .. .. .. .. .. .. .. .. .. 344

IV.

Farther Observations on the Lateral or Serpentine Curvature of the Spine, and on the Treatment of Contracted Limbs. By John Shaw, Surgeon to the Middlesex Hospital. .. .. .. 366

V.

An Essay on Tetanus, founded on Cases and Experiments. By Joseph Swan, M.R.C.S. .. .. .. .. .. .. .. .. 387

VI.

Elements of Operative Midwifery, comprising a Description of certain new and improved Powers for assisting difficult and dangerous Labours. By David D. Davis, M.D. .. .. .. 396

VII.

Mr Carmichael's Essay on Venereal Diseases, and the Uses and Abuses of Mercury in their Treatment. .. .. .. .. .. 435

VIII.

Instructions to Mothers and Nurses on the Management of Children in Health and Disease. By JAMES KENNEDY, M.D.. . 485

IX.

Clinique Medicale, ou Choix d'Observations recueillies à la Clinique de M. Lerminier, Médecin de l'Hôpital de la Charité, &c. Par G. ANDRAL, Fils: Deuxieme Partie—MALADIES DE POITRINE .. .. .. .. .. .. .. .. .. .. 493

X.

### Quarterly Periscope

OF

### PRACTICAL MEDICINE;

BEING

*THE SPIRIT OF THE MEDICAL JOURNALS,*

FOREIGN AND DOMESTIC.

Preface to the Periscope .. .. .. .. .. .. .. .. .. 523

I. PATHOLOGY.

1. Hospital Reports of M. M. Recamier and Martinet from the Hotel Dieu, for the first quarter of 1825 :
    1. Fever .. .. .. .. .. .. .. .. .. .. .. 525
    2. Diseases of the Encephalon. . .. .. .. .. .. .. 526
    3. Diseases of the Chest .. .. .. .. .. .. .. .. 527
    4. Abdominal Inflammations .. .. .. .. .. .. .. 528
    5. Colica Pictonum .. .. .. .. .. .. .. .. .. 528
2. Dr. Baker's Case of Organic Disease of the Heart, &c. . .. .. 529
3. M. Andral, Jun. on the State of the Stomach in Phthisis .. 530
4. Mr. Torbet, Dr. Duncan, and Dr. Kennedy, on Intestinal Calculus .. .. .. .. .. .. .. .. .. .. .. .. 532
5. Dr. Brandreth on Hydrophobia .. .. .. .. .. .. .. 534
6. Dr. Howison's Case of Enlarged Heart .. .. .. .. .. .. 536

II. SURGERY.

1. Dr. Fuschetus' Case of Gastrotomy for Intus-susception .. 537
   Remarks on this Operation, with the Case of Mrs. Belzoni's Servant .. .. .. .. .. .. .. .. .. .. .. .. 538
2. Mr. Averill's Case of Lithotomy .. .. .. .. .. .. .. 539
3. Mr. Davies' Case of Phlegmasia Dolens requiring Amputation 540
   Dr. Davis's Case of ditto in a Male .. .. .. .. .. .. 541
4. Dr. Jackson's Case of Paracentesis Thoracis. . .. .. .. .. 542

5. Mr. Pettigrew on Extirpation of the Tonsils .. .. .. 545
6. Mr. Symes on the Treatment of Incised Wounds.. .. .. .. 545
7. Mr. Allan on Perforations of the Soft Palate .. .. .. 547
8. Mr. White on Sympathetic Irritation of the Urethra .. .. 549
9. On Tracheotomy in Diseases of the Larynx.. .. .. .. 549
10. Dr. Cumin on the Treatment of Ganglion .. .. .. .. 551
11. Professor Walther on Amputation at the Hip-joint, with an Answer to his Strictures on Mr. Guthrie's Operation .. .. 551
12. On Herpes Genitalis .. .. .. .. .. .. .. .. 556
13. Remarkable Case of Idiotism cured by Extirpation of the Clitoris .. .. .. .. .. .. .. .. .. .. .. 557
14. The Copenhagen Needle Patient .. .. .. .. .. .. .. 559
15. Dr. Webster on Acupuncturation.. .. .. .. .. .. 562

### III. THERAPŒIA.

1. On Chorea .. .. .. .. .. .. .. .. .. .. .. .. 563
2. Recovery from taking Arsenic and Sublimate.. .. .. .. 565
3. Mr. Rennie on the Treatment of Scrofulous Ulcers.. .. .. 565
4. Dr. Gibson on Mercurial Fumigation .. .. .. .. .. 568
5. Dr. Belcher on the Treatment of Yellow Fever .. .. .. 568
6. Remedies for the Itch .. .. .. .. .. .. .. .. .. 570
7. Dr. Booth on Hydrophobia .. .. .. .. .. .. .. 571
8. Dr. Foucart on the Utility of Sanguineous Evacuations in the Diseases of Old People.. .. .. .. .. .. .. .. 571
9. Dr. Henry Davies on the Treatment of Hydrocephalus .. .. 571

### *Pharmacology.*

1. Mr. Frost on the Flowers of Colchicum.. .. .. .. .. 573
2. Mr. Wood on large Doses of Tartar Emetic .. .. .. .. 573
3. Cheap Mode of preparing Sulphate of Quinine .. .. .. 574
4. Dr. Milne on Pomegranate Root in expelling Tænia .. .. 574
5. Dr. Brandreth on Injection of Acetate of Morphia in Hydrophobia .. .. .. .. .. .. .. .. .. .. .. 575
6. Mr. Cox on Oil of Turpentine in Erysipelas .. .. .. .. 576
7. Mr. Annesley on the Action of Calomel in Indian Diseases .. 576

### IV. MEDICAL JURISPRUDENCE.

1. An Enquiry into the History of the Belief in Contagion.. .. 578
2. Coroner's Inquest at St. George's Hospital .. .. .. .. 582
Remarks on the Letter of "a disinterested Physician" (Med. Repos.) .. .. .. .. .. .. .. .. .. .. 585
3. Sir Henry Halford's Oration on Opening the New College of Physicians, in Pall Mall East, June 25th, 1825 .. .. .. 585
Eloquent Eulogy on the Character of Dr. Baillie .. .. 585

### XI.

### ANALECTA MINORA.

1. Dr. Milne on the Application of Leeches .. .. .. .. 587

2. Old Operations for Ovarian Dropsy .. .. .. .. 588

3. Corpora Lutea in a Child .. .. .. .. .. .. 588

4. New Mode of preparing Mercurial Ointment.. .. .. .. 588

5. Antidotes .. .. .. 589

6. Euphorbia Lathyris, or Caper Spurge .. .. .. .. 589

7. Variolous Eruption.. .. .. .. .. .. .. .. 589

8. Dr. W. Philip's Letter to the Editor .. .. .. .. .. 589

### XII.

Bibliographical Record .. .. .. .. .. .. .. .. .. 591

### XIII.

### EXTRA-LIMITES.

I. Dr. Gordon Smith's Reclamation .. .. .. .. .. 596
II. Dr. Davis on the Forceps .. .. .. .. .. 597
III. General Meeting of the Associated Apothecaries .. .. 598

### XIV.

### INTELLIGENCE, CORRESPONDENCE, &c.

Anatomie Vivante, or Skeleton Importation Company.. .. .. 599
Dr. Heiniker.. .. .. .. .. .. .. .. .. .. .. 600
Sea Water Baths .. .. .. .. .. .. .. .. .. .. 601
Additional Subscribers .. .. .. .. .. .. .. .. 602

